# Combining Endocrine Therapy with Trastuzumab Emtansine Improves Progression-Free Survival and Overall Survival in HER2-Positive, Hormone Receptor-Positive Metastatic Breast Cancer

**DOI:** 10.3390/medicina60060951

**Published:** 2024-06-07

**Authors:** Oğuzcan Kınıkoğlu, Hatice Odabas, Yunus Emre Altıntaş, Anıl Yıldız, Burçin Çakan, Goncagül Akdağ, Sedat Yıldırım, Hamit Bal, Tuğba Kaya, Salih Tünbekici, Deniz Işık, Tuğba Başoğlu, Mahmut Emre Yıldırım, Nedim Turan

**Affiliations:** 1 Department of Medical Oncology, Health Science University, Kartal Dr. Lütfi Kirdar City Hospital, Istanbul 34865, Turkey; odabashatice@yahoo.com (H.O.); yunusaltintas1688@gmail.com (Y.E.A.); akdaggoncagul@gmail.com (G.A.); rezansedat@hotmail.com (S.Y.); hamitbal@yahoo.com (H.B.); tugbakaya89@hotmail.com (T.K.); dnz.1984@yahoo.com (D.I.); basoglutugba@gmail.com (T.B.); emremahmutyildirim@gmail.com (M.E.Y.); turan.nedim@hotmail.com (N.T.); 2 Department of Medical Oncology, Istanbul University Oncology Institute, Istanbul 34093, Turkey; anilyildiz@live.com; 3 Department of Medical Oncology, Bağcılar Research and Training Hospital, Istanbul 34212, Turkey; burcin.cakandemirel@gmail.com; 4 Department of Medical Oncology, Ege University Faculty of Medicine, Izmir 35100, Turkey; slhtnbkc@yahoo.com

**Keywords:** metastatic breast cancer, trastuzumab emtansine, endocrine therapy, pertuzumab plus trastuzumab

## Abstract

*Background and Objectives:* Patients with human epidermal growth factor receptor 2 (HER2) -positive, hormone receptor-positive (HR-positive) metastatic breast cancer (MBC) usually undergo trastuzumab emtansine (T-DM1) therapy in subsequent lines. Combining endocrine therapy (ET) with T-DM1 can improve treatment outcomes in this subtype. Therefore, this study aimed to investigate the benefits of using T-DM1 with ET in HER2-positive and HR-positive MBC. This study was the first to investigate the benefits of combining ET with T-DM1. *Material and Methods:* This study analyzed the medical records of patients with HER2-positive and HR-positive MBC who were treated with T-DM1 from June 2010 to December 2021. The patients were divided into groups based on whether they received concomitant ET with T-DM1. The primary endpoint was to determine the progression-free survival (PFS), while the secondary endpoints were overall survival (OS), objective response rate, and safety of the treatment. *Results:* Our analysis examined 88 patients, of whom 32 (36.4%) were treated with T-DM1 in combination with ET. The combination therapy showed a significant improvement in median PFS (15.4 vs. 6.4 months; *p* = 0.00004) and median OS (35.0 vs. 23.1 months; *p* = 0.026) compared to T-DM1 alone. The ORR was also higher in the combination group (65.6% vs. 29.3%; *p* = 0.026). Patients treated with pertuzumab priorly had reduced median PFS on T-DM1 compared to those who were not treated with pertuzumab (11.7 vs. 5.4 months, respectively; *p* < 0.01). T-DM1 demonstrated better median PFS in HER2 3+ patients compared to HER2 2+ patients, with an amplification ratio of >2.0 (10.8 vs 5.8 months, respectively; *p* = 0.049). The safety profiles were consistent with previous T-DM1 studies. *Conclusions:* The combination of T-DM1 with ET can significantly improve PFS and OS in patients with HER2-positive and HR-positive MBC. Our study suggests that prior pertuzumab treatment plus trastuzumab treatment might decrease T-DM1 efficacy.

## 1. Introduction

Human epidermal growth factor receptor 2 (HER2) -positive breast cancers, characterized by the overexpression of the HER2 receptor, constitute approximately 15–20% of all breast cancers and generally demonstrate aggressive tumor behavior [1]. Introducing HER2-targeted therapies has revolutionized the treatment landscape for HER2-positive breast cancers. Currently, trastuzumab and pertuzumab combined with chemotherapy are the standard therapy for first-line HER2-positive metastatic breast cancer (MBC) [2].

Further innovation has come with antibody–drug conjugates, where potent cytotoxic agents are directly linked to HER2-targeting antibodies. Trastuzumab emtansine (T-DM1) combines the HER2-specific effects of trastuzumab with the microtubule-inhibitory action of emtansine. Upon binding to HER2, the T-DM1 complex undergoes internalization and lysosomal degradation, enabling the targeted intracellular delivery of the cytotoxic payload for enhanced tumor-cell-killing while potentially reducing systemic toxicity [3]. This strategy has demonstrated superiority over alternative therapies in trials such as EMILIA, solidifying its role in patients with HER2-positive MBC who have previously progressed on a trastuzumab-based regimen [4].

Within the HER2-positive category, a distinct subset co-expresses hormone receptors (HR) (estrogen receptor [ER] and/or progesterone receptor [PR]) and is classified as HER2-positive, HR-positive MBC [5]. Approximately half of HER2-positive breast cancers are HR-positive [6]. This accounts for nearly 10% of all breast cancers [7]. This subtype presents unique biological characteristics that influence treatment strategies and clinical outcomes. While targeted therapies such as T-DM1 have revolutionized patient outcomes in the broader HER2-positive population, achieving optimal results in the HR-positive subset remains an ongoing pursuit. This dual characteristic presents both an opportunity and a challenge. Targeting the HER2 pathway with T-DM1 holds promise, but the continued presence of HR suggests a potential benefit from incorporating endocrine therapy (ET) into the treatment regimen. The EGF30008 study focused on HR-positive MBC. Within the subgroup of patients who were also HER2-positive, adding lapatinib to letrozole significantly improved PFS and clinical benefit rates [8]. Other studies have also shown the benefit of adding ET to anti-HER2 therapy [9,10]. In first-line MBC, the superiority of trastuzumab plus pertuzumab and aromatase inhibitor (AI) over trastuzumab and AI in HR-positive, HER2-positive patients was demonstrated in the Pertain study [11]. Retrospective real-world data also support the addition of ET to dual anti-HER2 therapy in the first-line setting, which was found to prolong both PFS and overall survival (OS) [12]. These studies have shown that adding anti-HER2 therapy to ET effectively prolongs PFS and OS. However, it is unknown how adding ET to T-DM1 affects PFS and OS in MBC. Therefore, this study investigated the effectiveness of ET combined with T-DM1 in MBC.

## 2. Materials and Methods

### 2.1. Study Design and Patient Selection 

**Figure 1:**
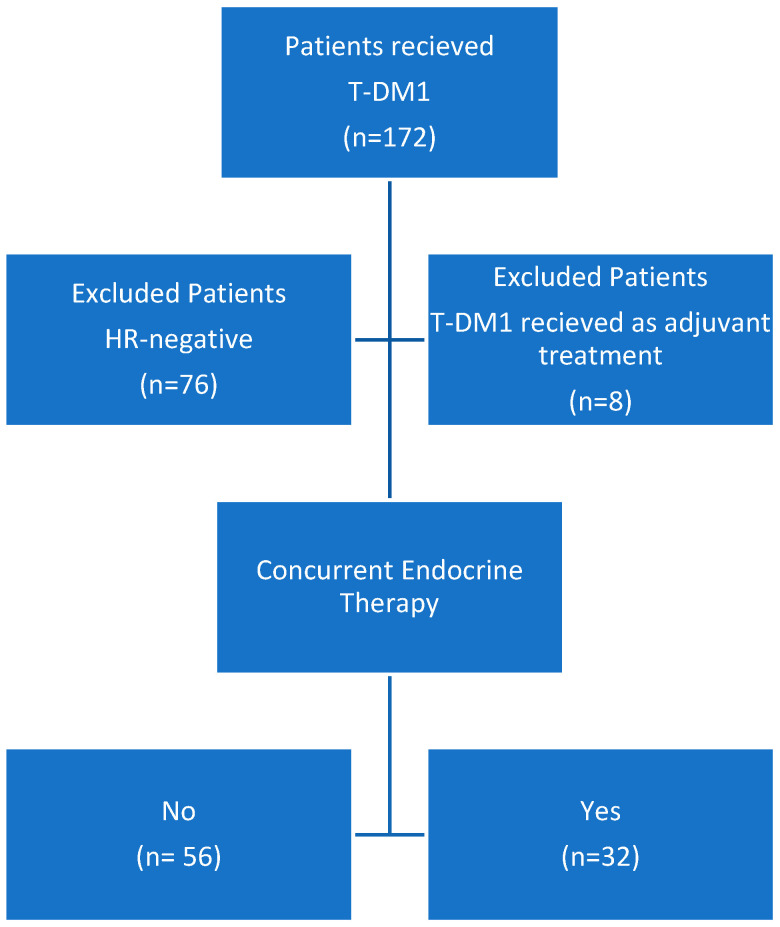
Flow chart describing patients receiving T-DM1 for HER2-positive MBC. Abbreviations: T-DM1, trastuzumab emtansine;; HR, hormone receptor.

This study was designed as a retrospective review of the medical records of patients with MBC who received T-DM1 therapy between June 2010 and December 2021 at our institution. Patients with HR-positive and HER2-positive MBC were selected, and those treated with T-DM1 were identified. Patients were divided into two groups according to whether they received ET along with T-DM1 or not (Figure 1). Patients were eligible for the study if they were female, 18 years or older, had an ejection fraction of ≥50%, and had radiologically confirmed HER2-positive, HR-positive MBC.

HER2-positive status was assessed using immunohistochemical (IHC) analysis (with 3+ indicating a positive status), fluorescence in situ hybridization (2+ with an amplification ratio of >2.0 indicating a positive status), or both. According to the American Society of Clinical Oncology/College of American Pathologists Guideline, tumors are defined as HR-positive when an IHC shows an Allred score of ≥3 for estrogen and progesterone receptors [13].

Clinical and pathological stages, prior treatments, metastatic organ sites, details of T-DM1 treatment regimens (dosing, schedule, and duration), the use of concomitant ET, and treatment outcomes were collected from medical records. Patients received T-DM1 at a dose of 3.6 mg/kg intravenously every 21 days. As an anti-hormonal treatment, either letrozole (2.5 mg/day), anastrozole (1 mg/day), exemestane (25 mg/day), or tamoxifen (20 mg/day) were given every day to the combination group according to doctors’ preferences. For perimenopausal patients, gonadotropin-releasing hormone analogs were given either monthly or every three months.

### 2.2. Assessments

The primary endpoint of this study was PFS, and the secondary endpoints were OS, objective response rate (ORR), and safety. Responses to T-DM1 treatment were assessed every three cycles until disease progression, death, or loss of follow-up occurred for patients who discontinued treatment for any other reason according to the *Response Evaluation Criteria in Solid Tumors* [14]. PFS was calculated as the months from T-DM1 treatment to disease progression or death (whichever occurred first). OS was defined as the time in months from T-DM1 initiation until death. Adverse events were graded during follow-ups according to the common terminology criteria for adverse events (version 3.0) [15].

### 2.3. Statistical Analysis

The survival data analysis estimated the 95% confidence intervals (CI) using the exact method. Either chi-square or Fisher’s exact tests were employed to assess the clinical and pathological characteristics of the patients. A *p*-value of <0.05 was considered statistically significant. Kaplan–Meier survival curves and log-rank tests were used to analyze PFS and OS. Univariate and multivariate Cox proportional hazards models were implemented to pinpoint factors associated with PFS and OS. Variables with *p*-values of <0.10 in the univariate analysis and factors that may have contributed to survival were included in the multivariate analysis. Missing data were excluded from the survival analysis. A statistical analysis was performed using SPSS Statistics 26.0 (IBM corporation, New York, NY, USA). A priori sample size analysis was conducted to define the appropriate number of patients for the study. The alpha value was determined as 0.05, the beta value as 0.20, and the effect size as 0.5 (large). After the analysis, the minimum number of patients needed to conduct this study was found to be 39 (using G*Power 3.1.9.7). In this study, we reached a total sample size of 88 patients, which exceeded our minimum calculated sample size.

### 2.4. Ethical Statement

This study was performed in accordance with the principles of the Declaration of Helsinki and approved by Kartal Dr. Lütfi Kırdar City Hospital’s Ethics/Institutional Review Board (date: 27 March 2024, no: 2024/010.99/2/11).

## 3. Results

### 3.1. Study Population and Disease Characteristics

This study analyzed 88 patients diagnosed with HR-positive, HER2-positive MBC between June 2010 and December 2021. The median patient age was 47.5 years (range = 25–82). Forty-seven patients (53.4%) were premenopausal, and seventy had ECOG PS values of zero. A majority were both estrogen and progesterone receptor-positive (85.2%). At the time of T-DM1 treatment, 63.6% had two or more metastases, while 69.3% had visceral disease. Notably, 52 patients (59.1%) received T-DM1 as a second-line treatment, with the remainder receiving it as a third-line treatment or later option. Out of 88 patients, 35 (39.8%) had received pertuzumab before. Only one patient received pertuzumab in the neoadjuvant setting, while 34 received it in their first-line treatments. Among the patients, 32 (36.4%) received concomitant ET with T-DM1. Of those who received ET, 62.5% received AI, 25% received tamoxifen, and 12.5% received fulvestrant. The two groups had no significant differences regarding their baseline characteristics (Table 1).

### 3.2. Efficacy

**Figure 2:**
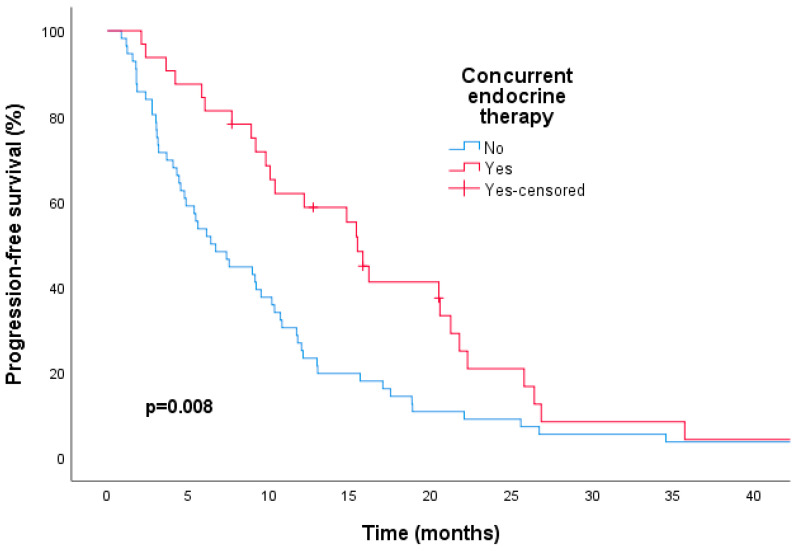
Kaplan–Meier curves indicating a significant improvement in progression-free survival when endocrine therapy was combined with trastuzumab emtansine compared to trastuzumab emtansine alone.

**Figure 3:**
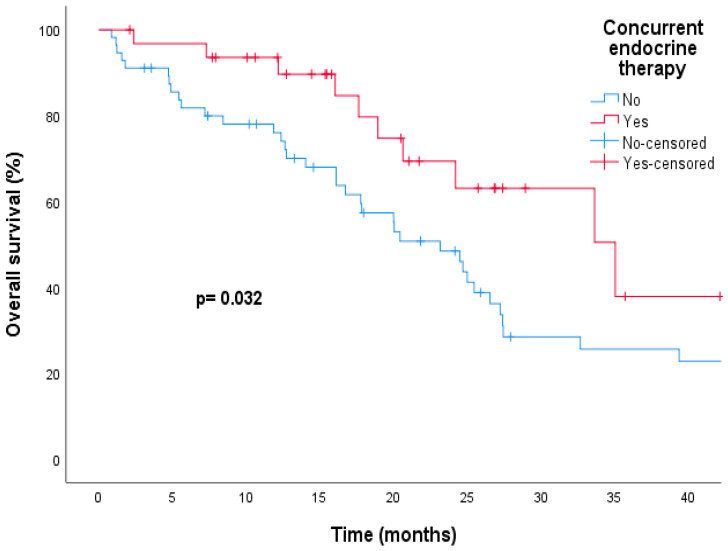
Kaplan–Meier curve indicate a significant improvement in overall survival when endocrine therapy was combined with trastuzumab emtansine compared to trastuzumab emtansine alone.

The median duration of follow-up was 22.1 months (range = 0.8–88.8). By the time data were collected, 47 (53.4%) patients had passed away and 83 patients (94.3%) had experienced disease progression. The entire group had a median PFS of 9.5 months (95% confidence interval [CI], 8.1–10.8) and a median OS of 25 months (95% CI, 21.2–28.7). Patients receiving T-DM1 in the second line setting had a median mPFS of 9.2 months (95% CI, 7.5–10.8) and a median mOS of 24.7 months (95% CI, 19.8–29.5). We investigated the factors contributing to PFS and OS using univariate and multivariate analyses (Table 2 and Table 3). The combination of T-DM1 and ET significantly increased the median PFS compared to those who received T-DM1 alone (15.4 vs. 6.4 months, respectively; hazard ratio [HR] of 0.54; 95% CI, 0.34–0.85; *p* = 0.008), and this remained significant in the multivariate analysis (HR, 0.31; 95% CI, 0.18–0.53; *p* = 0.00004) (Table 2, Figure 2). The addition of ET was also associated with a statistically significant improvement in median OS compared to the T-DM1 alone group (35.0 vs. 23.1 months, respectively; HR, 0.47; 95% CI, 0.23–0.95; *p* = 0.037), and this was confirmed in the multivariate analysis (HR, 0.44; 95% CI, 0.21–0.90; *p* = 0.026) (Table 3, Figure 3).

**Figure 4:**
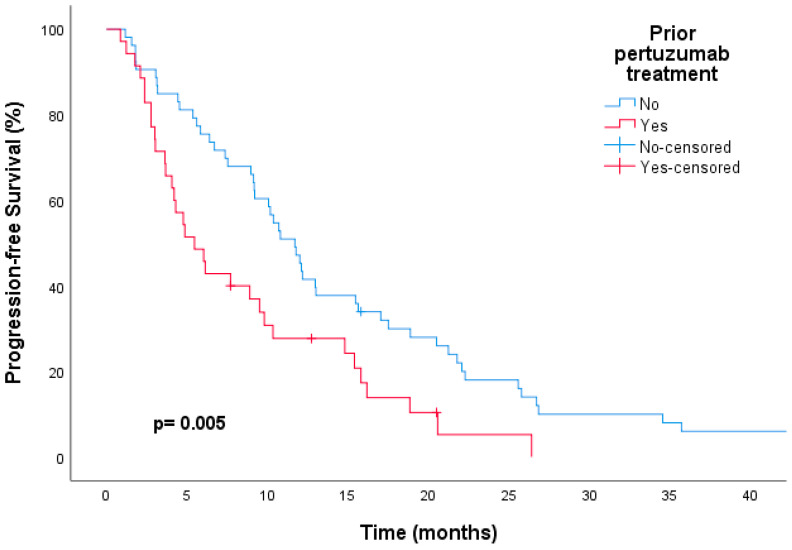
Kaplan–Meier curves showing longer progression-free survival with trastuzumab emtansine for patients not treated with pertuzumab compared to those treated with pertuzumab.

**Figure 5:**
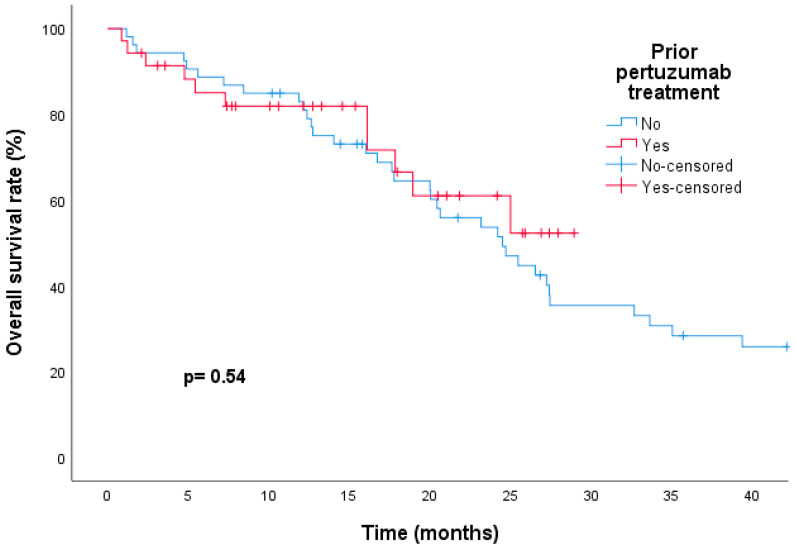
Kaplan–Meier curves showing similar overall survival with trastuzumab emtansine treatment for patients previously treated with pertuzumab compared to those treated without pertuzumab.

It was also found that patients who did not receive pertuzumab before T-DM1 treatment had significantly longer median PFS rates than those who were treated with pertuzumab before T-DM1, regardless of ET. This was observed in both the univariate (11.7 vs. 5.4 months, respectively; HR, 0.52; 95% CI, 0.32–0.83; *p* = 0.006) and multivariate (HR, 0.43; 95% CI, 0.24–0.75; *p* = 0.005) analyses (Figure 4). The median OS of patients who had previously received pertuzumab was not reached, while the median OS of patients who had not received pertuzumab was 24.5 months. However, there was no statistically significant difference in mOS between the two groups (not available [NA] versus 24.5 months, respectively; HR, 0.80; CI, 0.40–1.61; *p* = 0.54) (Figure 5).

**Figure 6:**
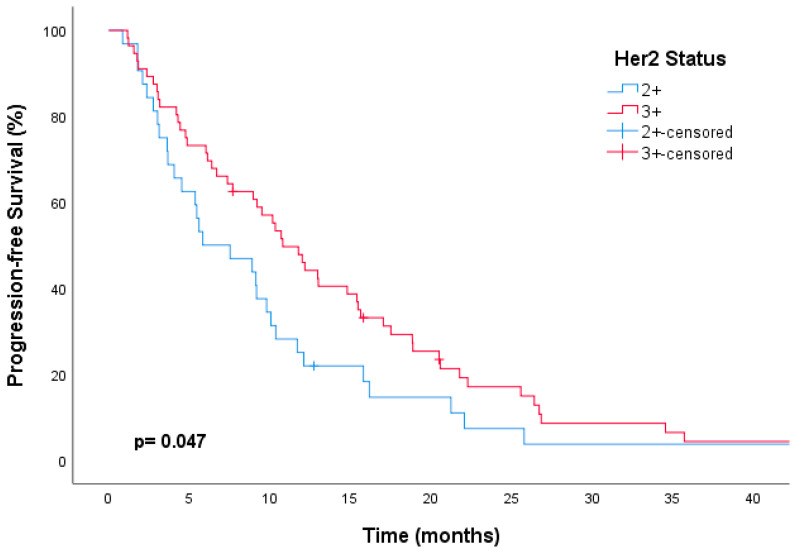
Kaplan–Meier curves showing improved progression-free survival with trastuzumab emtansine treatment for HER2 3+ metastatic breast cancer compared to HER2 2+, with an amplification ratio of >2.0 (positive status).

When the patients were examined according to their HER2 status, those with HER2 3+ had higher mPFS rates than those with HER2 2+ (with an amplification ratio of >2.0 indicating positive status) (10.8 vs. 5.8 months, respectively; HR, 0.63; 95% CI, 0.40–0.99; *p* = 0.049) (Figure 6). This was also significant in the multivariate analyses (HR, 0.52; 95% CI, 0.32–0.83; *p* = 0.01). However, although a numerically better median OS was found in the HER2 3+ patients, no statistical difference was found (25.0 vs. 20.0 months; HR, 0.89; 95% CI, 0.49–1.62; *p* = 0.47) (Table 3).

When we looked at the number of metastatic sites before T-DM1 treatment, the median PFS of the patients with lower numbers of metastatic sites was significantly higher in the univariate (15.6 vs. 7.5 months, respectively; HR, 0.34; 95% CI, 0.20–0.57; *p* = 0.0001) and multivariate analyses (HR, 0.27; 95% CI, 0.14–0.51; *p* = 0.001), and this significance was accompanied by the median OS in both the univariate (35.0 vs. 20.0 months, respectively; HR, 0.41; 95% CI, 0.21–0.77; *p* = 0.006) and multivariate analyses (HR, 0.37; 95% CI, 0.18–0.76; *p* = 0.003) (Table 3).

Upon examining the number of T-DM1 treatment cycles received by the patients who did and did not receive ET, it was observed that the patients who received ET could undergo more T-DM1 treatments. The median cycles of T-DM1 treatment were 16 (range = 4–99) in the ET group and 9 (range = 1–80) in the non-ET group. In the combined treatment group, the ORR was 65.6% and the disease control rate (DCR) was 71.9%, revealing a statistically significant difference in ORR in favor of the combined treatment group compared to the T-DM1 alone group (*p* = 0.026) (Table 4).

In terms of toxicity, the grades 3 and 4 adverse-effect profiles showed thrombopenia in five patients (6%), decreased ejection fractions in three patients (3%), and increased liver enzymes in three patients (3%). In total, 13% of our patients developed grades 3 and 4 side effects, and the treatments were discontinued in seven patients (8%) due to side effects (Table 5).

## 4. Discussion

This study was the first to examine the effectiveness of combining T-DM1 with ET in treating HER2-positive and HR-positive MBC. The rationale for combining T-DM1 with ET therapy stemmed from the unique biological characteristics of HER2-positive and HR-positive breast cancer. HER2 overexpression promotes uncontrolled cell growth and survival [16], while the presence of HR indicates the potential dependence of cancer cells on estrogen- and/or progesterone-signaling [17]. T-DM1 effectively targets HER2-positive cancer cells, but it may not fully address the contribution of HR-signaling to tumor growth and survival in this specific patient population. This is because HER2-positive cancers can develop resistance to anti-HER2 therapies by activating alternative growth pathways, such as the ER pathway [18]. The benefits of combination therapy can be attributed to several potential mechanisms. First, the combination reduces tumor burden by inhibiting the growth of HR-positive cancer cells, potentially shrinking tumors and delaying their progression. Additionally, it may prevent or delay the development of resistance to anti-HER2 therapies. This is supported by evidence that ER-positive, HER2-positive cell lines treated with HER2 inhibitors often exhibit increased ER activity, indicating an adaptive survival mechanism that the combined therapy blocks [19]. Finally, the combination could offer synergistic effects with T-DM1 by simultaneously targeting multiple crucial pathways in cancer-cell growth and survival, potentially leading to a more potent anti-cancer effect than anti-HER2 therapy alone [18].

Our findings support the initial hypothesis, demonstrating a statistically significant improvement in median PFS (15.4 vs. 6.4 months; *p* = 0.008) and median OS (35.0 vs. 23.1 months; *p* = 0.037) for MBC patients receiving ET with T-DM1. ORRs were also significantly higher in the ET group than in the non-ET group (65.6% vs. 29.3%; *p* = 0.026). Our observations aligned with emerging data from clinical trials. For instance, the TANDEM trial showed that the combination therapy was significantly more effective than AI alone regarding PFS, ORR, time to progression, and clinical benefit rates [20]. The ALTERNATIVE trial found that lapatinib plus trastuzumab and an AI were more effective than trastuzumab plus an AI regarding PFS benefits [21].

Our study revealed a reduction in median PFS for T-DM1 following exposure to pertuzumab compared to the non-pertuzumab group (11.7 vs 5.4 months, respectively, respectively; *p* = 0.006) [4]. This finding was consistent with previous retrospective analyses [22,23,24]. For example, in the study by Fabi A et al., patients who received T-DM1 after pertuzumab plus trastuzumab had shorter PFS rates compared to those who received trastuzumab alone (8 vs. 12 months, respectively, respectively) [22]. In another study, the PFS rates were 2.7 months vs. 7.8 months in favor of trastuzumab compared to pertuzumab plus trastuzumab, respectively [24]. Our research did not reveal any significant impact of prior exposure to pertuzumab on the OS of patients who received T-DM1 treatments. It is worth mentioning that the median follow-up period for patients who received pertuzumab was 14.4 months (range of 0.8–28.9), which was shorter than the non-pertuzumab group, where the median follow-up period was 20.6 months (range = 1.1–88.8). Therefore, sufficient follow-up times were not achieved for calculating the median OS of these patients on T-DM1.

The SePHER study suggested that the diminished efficacy of T-DM1 may have resulted from the potent dual blockade of HER2 receptors by pertuzumab plus trastuzumab [25]. The inhibition of HER2 receptors at different binding sites may trigger compensatory adaptive responses in tumor cells, such as the downregulation of surface HER2 receptor expression. This reduced HER2 receptor availability likely impedes T-DM1 binding, hindering the efficient delivery of its cytotoxic payload and potentially contributing to its decreased efficacy [25]. Unique or overlapping resistant mechanisms specific to each HER2-targeted therapy might also have played roles in these outcomes [26].

While T-DM1 retains potential benefits for patients previously treated with pertuzumab plus trastuzumab, studies have suggested the possibility of reduced effectiveness. This highlights the complex challenges of managing HER2-positive MBC. It is crucial to acknowledge the limitations of retrospective studies. Rigorous prospective investigations are necessary to draw definitive conclusions about T-DM1’s efficacy following pertuzumab use.

Our observed mPFS disparity between HER2 2+ (fluorescence in situ hybridization with an amplification ratio of >2.0 indicating positive status) and HER2 3+ patients aligned with the hypothesis that downregulated HER2 expression reduces T-DM1 activity (5.8 vs. 10.8 months, respectively; *p* = 0.049). This suggests a potential correlation between HER2 expression levels and T-DM1 efficacy, similar to the trend observed by Tinterri et al. where triple-positive tumors showed better recurrence outcomes [27]. Patients with lower HER2 surface abundances might experience corresponding decreases in T-DM1-mediated PFS. Further research is needed to clarify the precise influence of HER2 expression levels and tumor biology on T-DM1 treatment outcomes.

The median PFS of our patients was comparable to that observed in the EMILIA trial (9.5 vs. 9.6 months, respectively), where the median PFS was 9.6 months for the T-DM1 arm and 6.4 months for the capecitabine-lapatinib combination arm (*p* < 0.001) [4]. Although we expected a higher median PFS due to the absence of hormone-negative patients in our cohort, a substantial number of patients who had previously received pertuzumab may have reduced the PFS of the entire group. Our patient population also included individuals who received T-DM1 therapy in third-line and above settings, unlike the EMILIA trial. Despite these potential drawbacks, adding ET appears to have compensated for these negative factors.

When we examined the side-effect profiles, adverse effects of grade 3 or above were observed in approximately 31.8% of the patients, and treatment was discontinued in seven patients (7.9%). Our toxicity profile was close to that of the Emilia trial, which reported 40.8% with grade 3 or above adverse events and 5.9% with treatment discontinuation [4]. The number of patients was insufficient to determine whether the side-effect profile was higher in the group receiving endocrine therapy. The similar side-effect profile and treatment discontinuations suggest that T-DM1 combined with ET treatment is a safe and viable option.

We also wanted to look for signs of endocrine resistance in the patients receiving T-DM1 who received prior treatment with endocrine therapy. However, we could not reach a sufficient number of patients to perform this analysis because most of our patients had already received ET in previous lines, and there were no patients who did not receive ET in the combined treatment group. This retrospective study design and the relatively limited number of patients are the weaknesses of this study. A more extensive, prospective study with a broader patient population would be necessary to understand this relationship fully.

## 5. Conclusions

This study is the first to highlight the value of combining T-DM1 with ET for treating HER2-positive and HR-positive MBC. Our findings demonstrate significant improvements in both PFS and OS with the combination therapy, supporting the rationale of dual targeting for more effective treatment. Our study suggests that prior pertuzumab plus trastuzumab treatment might decrease T-DM1 efficacy. Further research is crucial to confirm this finding and understand its mechanisms. Additionally, we observed a PFS difference in favor of the HER2 3+ group compared to the HER2 2+ (fluorescence in situ hybridization with an amplification ratio of >2.0 indicating positive) status group. These results underscore the complexities of managing HER2-positive MBC and the urgent need for continued investigation into optimal treatment strategies and the factors influencing drug response within this patient population. Our study had limitations that demand further investigation in larger prospective trials, including its retrospective nature, relatively small sample size, and short follow-up period.

## Figures and Tables

**Table 1:** Baseline patient characteristics by treatment group.

Characteristic	Total Cohort (n = 88)	With ET (n = 32)	Without ET (n = 56)	*p*-Value
Age (years)				
Median (range)	47.5 (25–82)	48.5 (31–82)	47.5 (25–74)	
Age groups, n (%)				0.70
<65	80 (90.9)	30 (93.7)	50 (89.3)	
≥65	8 (9.1)	2 (6.3)	6 (10.7)	
Menopausal status, n (%)				0.66
Pre-menopause	47 (53.4)	16 (50)	31 (55.4)	
Post-menopause	41 (47.6)	16 (50)	25 (44.6)	
ECOG PS, n (%)				0.79
0	70 (79.5)	25 (78.1)	45 (80.4)	
1	18 (20.5)	7 (21.9)	11 (19.6)	
Hormone receptor status, n (%)				0.94
ER-positive PR-positive	75 (85.2)	27 (84.4)	48 (85.7)	
ER-positive PR-negative	13 (14.8)	5 (15.6)	8 (14.3)	
HER-2 Status, n (%)				0.64
2+ *	32 (36.4)	13 (40.6)	19 (33.9)	
3+	56 (63.6)	19 (59.4)	37 (66.1)	
Metastatic at diagnosis, n (%)				0.11
Yes	39 (44.3)	18 (56.3)	21 (37.5)	
No	49 (55.7)	14 (43.7)	35 (62.5)	
Number of metastatic sites prior to T-DM1, n (%)				0.97
1	32 (36.4)	12 (37.5)	20 (35.7)	
≥2	56 (63.6)	20 (62.5)	32 (62.5)	
Visceral disease involvement, n (%)				0.63
Yes	61 (69.3)	21 (65.6)	40 (71.4)	
No	27 (30.7)	17 (34.4)	16 (28.6)	
Prior pertuzumab exposure, n (%)				0.07
Yes	34 (38.6)	17 (53.1)	18 (32.1)	
No	54 (61.4)	15 (46.9)	38 (67.9)	
T-DM1 line, n (%)				0.07
2	52 (59.1)	23 (71.9)	29 (51.8)	
≥3	36 (40.9)	9 (28.1)	27 (48.2)	
Progression, n (%)				0.057
Yes	83 (94.3)	28 (87.5)	55 (98.2)	
No	5 (5.7)	4 (12.5)	1 (1.8)	
Exitus status, n (%)				<0.01
Exitus	47 (53.4)	10 (31.2)	37 (66.1)	
Alive	41 (46.6)	22 (68.8)	19 (33.9)	

Abbreviations: ET, endocrine therapy; ECOG PS, Eastern Cooperative Oncology Group performance status; ER, estrogen receptor; PR, progesterone receptor; HER2, human epidermal growth factor receptor 2; T-DM1, trastuzumab emtansine. * Fluorescence in situ hybridization with an amplification ratio of >2.0 indicating positive status.

**Table 2:** Univariate and multivariate analyses for progression-free survival.

	Univariate Analysis			Multivariate Analysis	
Variable	mPFS (Months)	HR (95% CI)	*p*-Value	HR (95% CI)	*p*-Value
Age					
<65	9.5	0.87 (0.42–1.82)	0.72	0.75 (0.32–1.73)	0.50
≥65	9.1				
ECOG PS					
0	9.5	0.85 (0.50–1.44)	0.54	0.73 (0.39–1.35)	0.32
1	7.7				
HER-2 status					
2+ *	5.8				
3+	10.8	0.63 (0.40–0.99)	0.049	0.52 (0.32–0.83)	0.01
Visceral disease involvement					
Yes	9.1				
No	10.1	0.76 (0.47–1.2)	0.268	0.68 (0.37–1.23)	0.20
Number of metastatic sites prior to T-DM1, n (%)					
1	15.6	0.34 (0.20–0.57)	<0.01	0.27 (0.14–0.51)	<0.01
≥2	7.5				
Prior pertuzumab exposure					
Yes	5.4				
No	11.7	0.52 (0.32–0.83)	<0.01	0.43 (0.24–0.75)	<0.01
Concurrent ET					
Yes	15.4	0.54 (0.34–0.85)	<0.01	0.31 (0.18–0.53)	<0.01
No	6.4				

Abbreviations: mPFS, median progression-free survival; HR, hazard ratio; CI, confidential interval; ECOG PS, Eastern Cooperative Oncology Group performance status; HER2, human epidermal growth factor receptor 2; T-DM1, trastuzumab emtansine; ET, endocrine therapy. * Fluorescence in situ hybridization with an amplification ratio of >2.0 indicating positive status.

**Table 3:** Univariate and multivariate analyses for overall survival.

	Univariate Analysis			Multivariate Analysis	
Variable	mOS (Months)	HR (95% CI)	*p*-Value	HR (95% CI)	*p*-Value
Age					
<65	25.4	0.41 (0.19–0.90)	0.02	0.43 (0.16–1.19)	0.10
≥65	14.0				
ECOG PS					
0	27.2	0.51 (0.27–0.98)	0.04	0.67 (0.28–1.56)	0.35
1	17.8				
HER-2 status					
2+ *	20.0				
3+	25.0	0.89 (0.49–1.62)	0.47	0.68 (0.37–1.26)	0.22
Visceral disease involvement					
Yes	24.2				
No	33.6	0.55 (0.28–1.1)	0.08	0.87 (0.41–1.83)	0.72
Number of metastatic sites prior to T-DM1					
1	35.0	0.41 (0.21–0.77)	<0.01	0.41 (0.18–0.92)	<0.01
≥2	20.0				
Prior pertuzumab exposure					
Yes	NA	0.80 (0.40–1.61)	0.54	0.74 (0.35–1.58)	0.44
No	24.5				
Concurrent ET					
Yes	35.0	0.47 (0.23–0.95)	0.03	0.04 (0.22–0.95)	0.03
No	23.1				

Abbreviations: mPFS, median progression-free survival; HR, hazard ratio; CI, confidential interval; ECOG PS, Eastern Cooperative Oncology Group performance status; HER2, human epidermal growth factor receptor 2; T-DM1, trastuzumab emtansine; NA, not available; ET, endocrine therapy. * Fluorescence in situ hybridization with an amplification ratio of >2.0 indicating positive status.

**Table 4:** Overall tumor response.

Tumor Response	With ET (n = 32)	Without ET (n = 56)	*p*-Value
ORR *	21 (65.6)	22 (29.3)	0.03
CR, n (%)	4 (12.5)	4 (7.1)	0.44
PR, n (%)	17 (53.1)	18 (32.1)	0.01
SD, n (%)	7 (21.9)	12 (21.4)	0.98
PD, n (%)	4 (12.5)	22 (29.3)	0.01
DCR †, n (%)	23 (71.9)	33 (58.9)	0.25

Abbreviations: ET, endocrine therapy; ORR, objective response rate; CR, complete response; PR, partial response; SD, stable disease; PD, progressive disease; DCR, disease control rate. *, objective response included CR or PR. †, disease control rate was defined as CR, PR, or SD.

**Table 5:** Grade 3 and 4 adverse events in total cohort.

Adverse Events	Number of Patients	% of Patients
Any grade 3 or above event	28	31.8
Specific events		
Neutropenia	2	2.2
Anemia	2	2.2
Thrombopenia	9	10.2
Elevated ALT and AST	5	5.6
Decreased EF	3	3.4
Treatment Cessation	7	7.9

Abbreviations: EF, ejection fraction; ALT, alanine aminotransferase; AST, aspartate aminotransferase.

## Data Availability

The data presented in this study are available on request from the corresponding author.
